# The Wisconsin Dental College

**Published:** 1884-06

**Authors:** Adolph Peterman, C. E. Hebbell

**Affiliations:** Frankfort–on–the–Main.


AMERICAN JOURNAL

OF

### DENTAL SCIENCE.

Vol. xviii. THIRD SERIES—JUNE 1884. No. 2.

ARTICLE I.

### THE WISCONSION DENTAL COLLEGE.

BY DR. ADOLPH PETERMAN, FRANKFORT–ON–THE–MAIN.

(Translated for the "American Journal of Dental Science" by C E. Hebbell.

A few years have scarcely passed since the trade in Doctor Diplomas has been checked by the conviction of John Buchanan, proprietor of the celebrated Doctor Factory at Philadelphia. This disgraceful business is again stirring itself, and then as well as now these worthless papers are sought to be disposed of mainly to persons residing in Europe. The following lines may be in place to serve as an explanation before this evil assumes too great dimensions.

The New York *Haatszeitung* No. 62, of March, 1881, writes:

"Wisconsin enjoys the possession of a Doctor Diploma Factory, though it be only a Dentist Factory. The Denta College at Delavan, Wisconsion, issues such Diplomas "in absentia" for $12. The entire College consists of a few poorly furnished rooms in the second story and the President of the same, who calls himself Doctor, is Mr. George Morrison, who is at the same time Secretary and Treasurer, and perhaps the entire faculty in person. Besides Morrison are two more 'Professors,' viz: John Morrison (son of the President) as Professor of Dental Surgery, and D. B. Devendorf, M. D., as Professor of Anatomy and Surgery."

Lately an advertisement was to be found in the columns of the "*Kladderadatsch*" and "*Fliegende Blatter*," offering the diploma of Doctor of Dental Surgery of an American College, and upon addressing the agent in England who would give all required information, the following answer was received:

WEST GRINSTEAD, *April 17th.* 1883.

*Dear Sir:*

In answer to your letter, I would inform you that I can provide you with the Diploma of Doctor of Dental Surgery from a Dental College in the State of Wisconsin (America.) The requirement is that you transmit in writing a work on Dental Science or Dental Mechanism. In America a person is at the same time Doctor of Dental Surgery and Dentist, upon request it may also be added that you have been appointed dentist. The price for this is 500 mark. If you are determined to procure for yourself this title, you will find me prepared to give all information and undertake the management. The Academy is authorized by the State to confer the Degree of Doctor, and the Diplomas are recognized in the United States.

Respectfully,
W. ROTT,
West Grinstead, Trussex, Eng.

In the last year this swindle Association known as the Wisconsin Dental College sent its proposals to all possible addresses in Germany. This *pure* College offers its Diplomas of Doctor of Dental Surgery for twelve dollars each, In order to disguise this gigantic swindle, these purchaseable and worthless diplomas are conferrcd "honoris causa." Investigations pending at the time between the Chancellor of the German Empire and American Embassy at Berlin, confirm the fact that American Universities and Colleges are not permitted to graduate "in absentia." As we now see this American law is evaded by the addition of "honoris causa."

The following is a copy of one of the said proposals which is generally accompanied by a printed request for an appointment is as follows:

This statement is made to the Faculty of the WISCONSIN DENTAL COLLEGE, located at Delavan, Walworth Co., Wisconsin, for the purpose of procuring an HONORARY Diploma and Degree of D. D. S., (Doctor of Dental Surgery.)

I am a regular Practicing Dentist.

I am a Resident of...... ...... ......................age...........

years. Have practiced Dentistry.................years.

Signed..................................

Witness............................ ......

Edward Hanft a manufacturer of artificial teeth was legally sentenced to pay a fine of 60 mark and to be imprisoned for twelve days, was requested to withdraw his sign and his so-called Diploma, suppressed for illegally bearing the title of Doctor with the addition of being a certified American Dentist by the *Schaffengericht* of Bremen, July 2nd, 1882. The said Hanft asserted that he considered himself justly entitled to the aforesaid titles having received a Diploma from the "Wisconsin Dental College." It was proven by the judicial acts that this College was a swindle concern whose only purpose was the sale of Doctor Diplomas. The German Counsel at Chicago judged the Wisconsin Dental College as follows: Unlimited freedom to exercise some trade or profession is in vogue there. Two or three persons can form a corporation and be entitled to all legal rights of the same. The President of the College, (Mr. Morrison), had informed him in reference to the accused Hanft; that the said Hanft had been especially recommended to him and his colleagues. Hanft had informed him (Morrison), that he had been practicing dentistry for five years in Europe upon which the honor of Doctor had been conferred upon him.

The State's Attorney had still further affirmed that Hanft had not employed himself with the treatment of diseased teeth for five years but that he had been engaged in the butter business with a merchant in Hanover, and that he had procured for himself upon the above mentioned plan without special examination upon untruthful assertions and for money a Diploma as Doctor which is without any value.

The Secretary of the Interior, and the Post Master General of the United States of North America have published the following in reference to the bogus transactions of the Wisconsin Dental College.

" Department of the Interior, Bureau of Education.

WASHINGTON, *July 22nd,* 1881.

To the Editor of the *Dental Cosmos:*

*Dear Sir:*—The attention of this Office having been repeatedly called to the fradulent practices of one Dr. George Morrison of the Dental College at Delavan, Wisconsin, which was noticed also in the *Dental Cosmos* of March, 1881, I took occasion early in July to call the attention of the Postmaster-General to the same, and I herewith inclose his reply which you may like to publish.

Very respectfully yours.

CHAS. WARREN, *Acting Commisssioner.*

---

Post-Office Department.

WASHINGTON *July 13th,* 1881.

THE HONORABLE THE SECRETARY OF THE INTERIOR.

*Sir:*—I have the honor to acknowledge the receipt of your communication of the 11th instant referring to this Department a communication from the Commissioner of Education, inclosing letters, etc., showing the fraudulent character of business carried on by Dr. George Morrison, President of Wisconsin Dental College, at Delavan, Wisconsin.

In reply I have to state that I have this day issued an order to the Postmaster at Delavan, Wisconsin, forbidding the payment of money orders or the delivery of registered letters to the said Dr. George Morrison.

Very respectfully,

THOS. L. JAMES, *Postmaster-General.*

In addition to the foregoing communications I can state with assurance that this Wisconsin Dental College has been repeatedly condemned in American Dental Periodicals for its nefarious transactions in Doctor Diplomas, for instance in the *Dental Cosmos* (Philadelphia, Chestnut and Twelfth Streets), 1881, pages 157, 490, 555—1884, pages 28, Johnston's *Dental Miscellaney*, (1260 Broadway, New York. 1881, pages 314, 349), which all published very complete communications about the swindling and shameful actions of Mr. Morrison and his so called Wisconsin Dental College.

